# Indirect effect of alpha-1-antitrypsin on endotoxin-induced IL-1β secretion from human PBMCs

**DOI:** 10.3389/fphar.2022.995869

**Published:** 2022-09-30

**Authors:** Sabina Janciauskiene, Srinu Tumpara, Nils Helge Schebb, Falk F. R. Buettner, Malwina Mainka, Kokilavani Sivaraman, Stephan Immenschuh, Veronika Grau, Tobias Welte, Beata Olejnicka

**Affiliations:** ^1^ Department of Respiratory Medicine, Biomedical Research in Endstage and Obstructive Lung Disease Hannover (BREATH), Member of the German Center for Lung Research (DZL), Hannover, Germany; ^2^ Department of Experimental Medicine, Lund University, Lund, Sweden; ^3^ Chair of Food Chemistry, Faculty of Mathematics and Natural Sciences, University of Wuppertal, Wuppertal, Germany; ^4^ Institute of Clinical Biochemistry, Hannover Medical School, Hannover, Germany; ^5^ Institute for Transfusion Medicine and Transplant Engineering, Hannover Medical School, Hannover, Germany; ^6^ Laboratory of Experimental Surgery, Department of General and Thoracic Surgery, Justus-Liebig-University Giessen, German Center for Lung Research, Giessen, Germany

**Keywords:** alpha-1-antitrypsin, PBMCs, LPS, Interleukin-1β, Cytokines, supernatants, mass-spectrometry, total thiols

## Abstract

Human alpha-1-antitrypsin (AAT) encoded by the *SERPINA1* gene, is an acute phase glycoprotein that regulates inflammatory responses *via* both protease inhibitory and non-inhibitory activities. We previously reported that AAT controls ATP-induced IL-1β release from human mononuclear cells by stimulating the release of small bioactive molecules. In the current study, we aimed to elucidate the identity of these putative effectors released from human PBMCs in response to AAT, which may inhibit the LPS-induced release of IL-1β. We pre-incubated human PBMCs alone or with different preparations of AAT (4 mg/ml) for 30 min at 37°C, 5% CO_2_, and collected cell supernatants filtered through centrifugal filters (cutoff 3 kDa) to eliminate AAT and other high molecular weight substances. Supernatants passed through the filters were used to culture PBMCs isolated from the autologous or a heterologous donors with or without adding LPS (1 μg/ml) for 6 h. Unexpectedly, supernatants from PBMCs pre-incubated with AAT (Zemaira^®^), but not with other AAT preparations tested or with oxidized AAT (Zemaira^®^), lowered the LPS-induced release of IL-1β by about 25%–60% without affecting *IL1B* mRNA. The reversed-phase liquid chromatography coupled with mass spectrometry did not confirm the hypothesis that small pro-resolving lipid mediators released from PBMCs after exposure to AAT (Zemaira^®^) are responsible for lowering the LPS-induced IL-1β release. Distinctively from other AAT preparations, AAT (Zemaira^®^) and supernatants from PBMCs pre-treated with this protein contained high levels of total thiols. In line, mass spectrometry analysis revealed that AAT (Zemaira^®^) protein contains freer Cys232 than AAT (Prolastin^®^). Our data show that a free Cys232 in AAT is required for controlling LPS-induced IL-1β release from human PBMCs. Further studies characterizing AAT preparations used to treat patients with inherited AAT deficiency remains of clinical importance.

## Introduction

Alpha-1-antitrypsin (AAT) is an acute phase glycoprotein and one of the most abundant protease inhibitors in human blood (1–2 g/L) (Carrell and Lomas, 2002; Janciauskiene et al., 2018). During the acute-phase reaction, in response to pro-inflammatory cytokines, specifically IL-6, but also IL-8, IL-17, and TGF-β, the plasma concentration of AAT can rise to 2-4-fold above the normal level (Gabay and Kushner, 1999). Although best recognized as an inhibitor of neutrophil elastase and proteinase 3, albeit with lower efficiency, AAT also inhibits other serine proteases like cathepsin G, thrombin, trypsin, and chymotrypsin (Janciauskiene et al., 2011). Most of these serine proteases target receptor proteins involved in cell signaling and pro-inflammatory cytokine expression, and act as activators of metalloproteases (Saunders et al., 2005; Austin et al., 2013). Therefore, AAT can modulate inflammatory responses indirectly, *via* serine protease inhibition (Liu et al., 2000). As a direct inhibitor of caspases, AAT also plays a role in cell apoptosis (Petrache et al., 2006; Toldo et al., 2011).

It is important to point out that AAT interacts with cytokines, free heme, heparins, lipoproteins, and other substances (Bergin et al., 2010; Karnaukhova et al., 2012). For example, AAT shows better anti-elastase activity in complex with high-density lipoprotein or heparin than by itself (Houghton et al., 2010; Lechowicz et al., 2020; Bai et al., 2022). AAT also interacts with chemoattractants, such as IL-8 and leukotriene B4 (LTB4), and thus modulates neutrophil adhesion and chemotaxis (Mcelvaney et al., 2020). The interaction between AAT and free heme neutralizes the cytotoxic effects of heme (Madyaningrana et al., 2021). These various interactions between AAT and its binding partners might dependent on the glycosylation and/or the hydrophilic/hydrophobic surface charges of the AAT (Mcelvaney et al., 2020). Notably, AAT contains one free cysteine residue at a position of 232 (Cys232). The thiol moiety of Cys232 is susceptible to oxidation under neutral pH conditions and is reactive with small molecules, such as cysteine, and glutathione (Griffiths et al., 2002; Fra et al., 2017; Luling et al., 2021).

Previous studies reported that AAT inhibits IL-1β release and/or expression *in vivo* and *in vitro* (Janciauskiene et al., 2004; Jonigk et al., 2013; Aggarwal et al., 2016; Joosten et al., 2016). IL-1β is one of the central cytokines associated with inflammation and innate immunity and is a stimulator of broad pro-inflammatory responses. Our current knowledge suggests that the activation of the cytosolic pro-inflammatory signaling complex (inflammasome) in human monocytes by various stimuli, including lipopolysaccharide (LPS), activates caspases, which cleave intracellularly preformed IL-1β (pro-IL-1β). The mature IL-1β is then released from the cells and activates the expression of other pro-inflammatory genes (Zhu and Kanneganti, 2017; Chan and Schroder, 2020). If the initial triggers of IL-1β remain, IL-1β may be self-propagating, thereby amplifying inflammation. Over the past years, diverse pharmacological substances have been reported to inhibit IL-1β release (Lopez-Castejon and Brough, 2011; Das et al., 2021). According to previous reports, preparations of AAT, which are used to treat emphysema patients with inherited AAT deficiency, can also lower IL-1β-related tissue damage by up-regulating IL-1 receptor antagonist, (Lewis et al., 2008; Abecassis et al., 2014). Concordantly, AAT as an inhibitor of caspase-3 may also restrict IL-1β release (Pott et al., 2009).

We have demonstrated that AAT inhibits the release of IL-1β in response to extracellular ATP (Siebers et al., 2018). Moreover, we provided evidence that AAT controls IL-1β maturation by activating calcium-independent phospholipase A2β and inducing the secretion of small anti-inflammatory molecule(s) from monocytic cells, which activate nicotinic acetylcholine receptors and thus prevent signaling of the ATP receptor P2X7 (Siebers et al., 2018). Calcium-independent phospholipase A2β preferentially cleaves phosphatidylcholines to free fatty acids and lysophosphatidylcholines (Scott et al., 2021). Free fatty acids can be further metabolized to diverse lipid mediators, including anti-inflammatory oxylipins, lysophosphatidylcholines and their metabolites, the potential agonists of unconventional nicotinic acetylcholine receptors expressed by monocytes and macrophages, which control the ATP-induced release of IL-1β (Grau et al., 2018; Siebers et al., 2018). The secretome of activated cells, like peripheral blood mononuclear cells (PBMCs) displays immunomodulatory and regenerative effects (Beer et al., 2015; Simader et al., 2019). Therefore, we wished to explore an idea that active anti-inflammatory lipid mediator(s) secreted from PBMCs after their pre-treatment with AAT may lower the LPS-induced IL-1β release, and aimed to identify these putative compounds.

## Materials and methods

### Alpha-1-antitrpysin preparations

AAT preparations Zemaira^®^ (CSL Behring, Kankakee, United States) and Prolastin^®^ (Grifols, Barcelona, Spain), which are both purified from human blood plasma, were used after buffer exchange to sterile phosphate-buffered saline (PBS) (20012-0191, Life Technologies, Bleiswijk, Netherlands) with centrifugal filter devices with a cutoff of 10 kDa (88517, Thermo Scientific, Rockford, United States). The protein concentration was determined using the BCA Protein Assay Kit (23227, Pierce™, Rockford, United States) according to the manufacturer’s protocol. We also used a glycosylated form of recombinant human AAT produced in Chinese hamster ovary cells (CHO-AAT) (gift from ExcellGene, Monthey, Switzerland) and human plasma AAT purified by affinity chromatography using AAT Select matrix (GE Healthcare Life Sciences, Cytiva, Sheffield, United Kingdom) according to the manufacturer’s recommendations. The oxidized AAT (oxAAT) was prepared from AAT (Zemaira^®^) by adding N-chlorosuccinimide (Sigma-Aldrich, Merck, Darmstadt, Germany) at a molar ratio of 1:20 (AAT: N-chlorosuccinimide) for 20 min at room temperature. Afterwards, to remove the N-chlorosuccinimide, AAT preparations were washed with PBS using viva spin-20 centrifugal filter devices with a cutoff of 10 kDa (Sartorius, Göttingen, Germany). The oxAAT did not form complexes with elastase and showed a retarded electrophoretic mobility relative to a native AAT (Moraga and Janciauskiene, 2000).

### Isolation of peripheral blood mononuclear cells

PBMCs were isolated from fresh peripheral human donor blood by using Lymphosep (PL-15-M, c.c.pro, Oberdorla, Germany) discontinuous gradient centrifugation as described elsewhere (Tumpara et al., 2021). Thereafter, cells we suspended in RPMI-1640 medium (21875-034, Life technologies, Bleiswijk, Netherlands) and used according to the experimental design. The PBMCs were isolated from healthy volunteers based on the ethical approval from Hannover Medical School (MHH-6895).

### Preparation of supernatants from peripheral blood mononuclear cells and experimental design

Total PBMCs (7.5 × 10^6^ per well) were plated to the non-adherent 6-well plate (657970, Greiner Bio-one, Kremsmünster, Austria), PBS or 4 mg/ml AAT in PBS was added, and cells were kept for 30 min at 37°C, 5% CO_2_. Afterwards, cells were spun down at 300 g for 5 min and cell-free supernatants were centrifuged using centrifugal filter devices with a cutoff of 3 kDa (88515, Thermo Scientific, Rockford, United States) to remove added AAT and other larger substances. Collected filtrates, named as basal supernatants [spn] or supernatants from cells pre-treated with AAT [spn-AAT], were used to culture adherent and total PBMCs (5 × 10^6^/per well) isolated from the same (autologous) or different (heterologous) donors. For all experiments, we cultured cells for 6 h in a basal medium [spn] or [spn-AAT] alone or with the addition of lipopolysaccharide (LPS, 1 μg/ml) (L2630, Sigma Aldrich, Darmstadt, Germany). At the end of the experiment, centrifuged cell culture supernatants we analyzed directly or stored at −80°C. The cells we separately collected and used for protein analyses.

### ELISA

Quantitative analysis of IL-1β, TNFα, IL-6, and IL-8 was performed by using ELISA kits purchased from R&D Systems (Minneapolis, United States) according to the manufacturer’s instructions (Table 1). Samples were analyzed using a microplate reader (Tecan Infinite M200, Männedorf, Switzerland). The absorbance was measured at 450 nm with the correction wavelength set at 540 or 570 nm.

**TABLE 1:** ELISA kit catalog numbers and assay sensitivity.

Cytokine/Chemokine	Catalog number	Assay range (pg/ml)
IL-1β	DY201	3.91–250
IL-6	DY206-05	9.38–600
IL-8	DY280	31.3–2,000
TNFα	DY210	15.6–1,000

### RNA isolation and real-time PCR

Total RNA was isolated with the RNeasy Mini kit (74106, Qiagen, Hilden, Germany) and quantified by using a NanoDrop spectrophotometer (Thermo Scientific, Bremen, Germany). For cDNA synthesis, 1 µg of total RNA was transcribed to cDNA by using a high-capacity cDNA reverse transcription kit (Thermo Fisher Scientific, Waltham, United States). Targeted genes were analyzed using TaqMan Gene Expression Assays (Thermo Fisher Scientific) on a StepOnePlus Real-Time PCR Systems machine (Thermo Fisher Scientific). All primers were purchased from Thermo Fisher Scientific (Table 2). The Ct value for each sample was calculated by determining the point at which the fluorescence exceeded a threshold limit. HPRT1 was used as a housekeeping gene in the same run. The measured gene expression was calculated according to the method 2^∆Ct^ (Ct value of target gene−Ct value of reference gene). All measurements were performed in duplicates.

**TABLE 2:** Primers used for gene expression analysis.

Target gene	Assay ID
*IL1B*	Hs01555410_m1
*IL6*	Hs00985639_m1
*CXCL8*	Hs00174103_m1
*TNFA*	Hs00174128_m1
*HPRT1*	Hs02800695_m1

### Western blotting

Cells were lysed in RIPA buffer (20 mM Tris-HCl pH 7.5, 150 mM NaCl, 9.5 mM EDTA, 1% Triton X-100, 0.1% SDS, and 1% sodium deoxy-cholate) (R0278, Sigma-Aldrich), supplemented with a protease inhibitor cocktail (P8340, Sigma-Aldrich). Equal amounts of lysed proteins, as determined by BSA protein assay kit (NOVAGEN, Darmstadt, Germany), were separated by 16.5% SDS-polyacrylamide gels prior to transfer onto polyvinylidene difluoride (PVDF) membranes (Millipore, Billerica, MA, United States). Membranes were blocked for 1 h with TBS + 0.01% Tween containing 5% low-fat milk powder (Roth, Karlsruhe, Germany) followed by overnight incubation at 4°C with primary mouse monoclonal anti-IL-β antibody (E7-2-hIL1β) (1:500, sc-32294, Santa Cruz, California, United States). For some experiments rabbit polyclonal antibody against total human blood plasma proteins were used (1:800, DAKO, Glostrup, Denmark) The immune complexes were visualized with horseradish peroxidase-conjugated antibodies (DAKO) and enhanced by ECL western blotting substrate (170-5060, Bio-Rad, California, United States). Images were taken by using the Chemidoc Touch imaging system (Bio-Rad, CA, United States).

### Analysis of oxylipins

Isolated PBMCs (10 × 10^6^ per ml) were incubated in RPMI medium or Hank´s balanced salt solution (HBSS) (14025092, Thermo Fisher Scientific) alone or with the addition of 4 mg/ml AAT (Zemaira^®^) for 30 min and collected supernatants were filtrated *via* 3 kDa cutoff centrifugal filters. For controls, under the same experimental conditions, RPMI medium and HBSS alone or with AAT (Zemaira^®^) supplementation were prepared. Two aliquots (each 500 µl) of the supernatants were prepared. One aliquot was transferred into a 2 ml reaction tube (72.695.500, Sarstedt, Nümbrecht, Germany) and after adding 500 µl of ice-cold LC-MS grade methanol was frozen in liquid nitrogen. A second aliquot was prepared in the same way but without freezing. All samples were analyzed according to the “standard operating procedure” for free oxylipins as described in detail previously (Mainka et al., 2020) by means of reversed-phase liquid chromatography coupled with tandem mass spectrometry. A spectrum of relevant oxylipins and multi-hydroxylated fatty acids was quantified. The list can be found in the Supplementary Table S1, including the limit of quantification in the medium.

### Total thiol assay

To detect free thiols (free cysteine, glutathione (GSH), and cysteine residues), we used a total thiol quantitation assay kit (5524, Amplite^®^ Fluorimetric Total Thiol Quantitation Kit, California, United States) according to the manufacturer’s recommendations. Briefly, 50 µl of freshly collected cell-free supernatants or AAT proteins, standard samples (range 0.12–30 µM), and blanks were added to the solid black 96-well plate (10421632, Fischer Scientific, Lund, Sweden). Afterwards, 50 µl of GSH working solution was added to each well and the plate was incubated in the dark for 25 min at room temperature. Fluorescence absorbance was measured by a microplate reader (Tecan Infinite M200), at Ex/Em = 490/525 nm (cutoff = 515 nm).

### Mass spectrometric analysis to detect free Cys232 in alpha-1-antitrypsin

Two microgram of AAT (Zemaira^®^ and Prolastin^®^) were separated by SDS-PAGE under non-reducing conditions and stained with Coomassie blue. Bands were cut out and digested overnight at 37°C with Trypsin Gold (Promega Corporation, Madison, United States) according to the manufacturer’s protocol with the important modification that the reduction step by dithiothreitol (DTT) was omitted. Thus only cysteine residues with free thiols should get carbamidomethylated (CAM-Cys) by iodoacetamide (IAA). Mass spectrometry was performed essentially as described previously (Cirksena et al., 2021) using a Linear Trap Quadrupole (LTQ) Orbitrap-Velos mass spectrometer equipped with a reversed-phase liquid chromatography system. Quantification of carbamidomethylation of Cys232 was performed essentially as described by Martin et al. (2016). MaxQuant (Tyanova et al., 2016) version 2.0.3.0 was used for automated peptide identification and calculation of “ratio mod/base” for CAM-Cys232 vs. unmodified Cys232. Preconfigured settings were used with the alteration that carbamidomethyl (C) was set as variable modification. A database only comprising the sequence of AAT without signal peptide was used to match mass spectra. In addition to the MaxQuant-based automated analysis, extracted ion-chromatogram (XIC)-based quantification was performed applying Xcalibur Qual Browser version 4.2.47 (Thermo Scientific, Waltham, MA, United States). XICs were generated based on the m/z values of the monoisotopic peptide ions using a mass tolerance of 20 ppm and a peptide mass accuracy of four decimal places for Cys232-containing peptides that had been identified by MaxQuant either with or without oxidation of Met226 and/or with or without carbamidomethylation of Cys232. From XICs, values for the peptide-specific peak areas were determined for each modification variant of the respective peptides.

### Statistical analysis

Data of ELISA and RT-qPCR were statistically analyzed using Sigma Plot 12.5 software package (Systat Software GmbH, Erkrathor, Germany). Graphical data presentation was performed using GraphPad. Prism [Version 9.1.2 (226), Dotamatics]. The Student’s *t*-test was applied to compare two sample means on one variable. When more than two groups were involved in the comparison, one-way ANOVA was used. The normally distributed data presented as a mean and standard deviation (SD). If the normality test failed, was performed the nonparametric Kruskal-Wallis one-way analysis followed by Mann-Whitney rank-sum test. A *p*-value below 0.05 considered as a significant.

## Results

### Peripheral blood mononuclear cells cultured in supernatants from autologous peripheral blood mononuclear cells pre-treated with alpha-1-antitrypsin (Zemaira^®^) release less IL-1β in response to lipopolysaccharide

**FIGURE 1:**
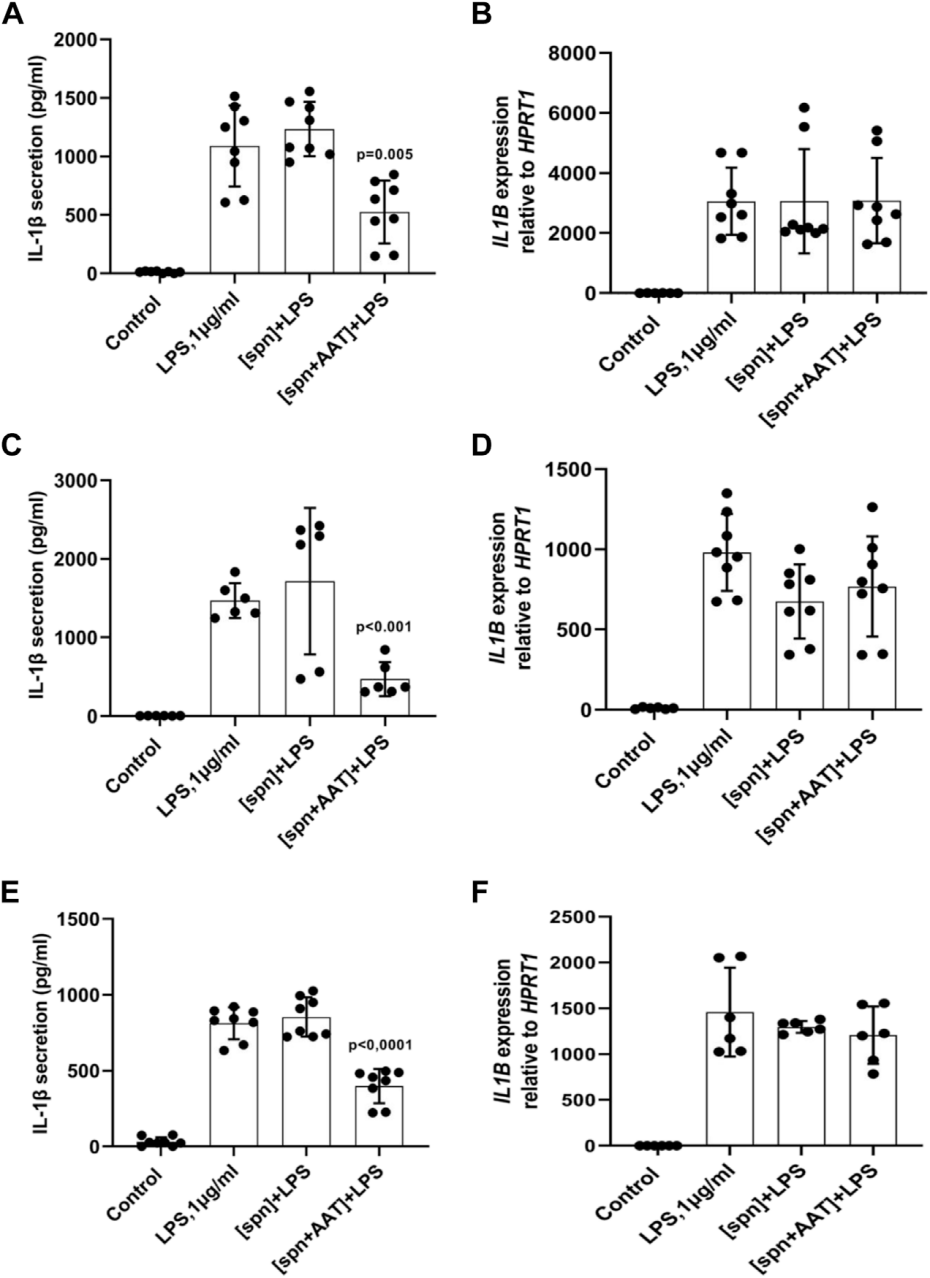
**(A–F)** Adherent **(A,B)** and total **(C,D)** autologous PBMCs, and total **(E,F)** heterologous PBMCs cultured in supernatants from pre-treated with AAT (Zemaira^®^) release less IL-1β, but do not change expression of *IL1B,* in response to LPS. LPS-induced IL-1β secretion and *IL1B* mRNA expression within 6 h in adherent **(A,B)** and total **(C,D)** human PBMCs cultured in supernatants from autologous PBMCs pre-treated for 30 min with PBS [spn] or with AAT (Zemaira^®^, 4 mg/ml), [spn + AAT]. LPS-induced IL-1β secretion **(E)** and expression **(F)** in total human PBMCs cultured for 6 h in supernatants from heterologous PBMCs pre-treated for 30 min with PBS [spn] or with AAT (Zemaira^®^, 4 mg/ml), [spn + AAT]. Each bar represents the mean (SD) from four or three independent donor experiments, with two repeats for each. *p*-value indicates significance between LPS and [spn + AAT]+LPS.

In the first set of experiments, PBMCs (5 × 10^6^ per well) were cultured in the plates for adherent cells alone or in the presence of LPS for 6 h in a regular medium or in the low molecular mass (<3 kDa) fraction of supernatants from autologous PBMCs pre-treated or not with AAT (Zemaira^®^, 4 mg/ml) for 30 min. As illustrated in Figure 1A, adherent PBMCs cultured in supernatants from AAT pre-treated cells [spn + AAT] released significantly less IL-1β in response to LPS than PBMCs cultured in supernatants from cells without AAT [spn] or in a regular medium. However, under all experimental conditions, we observed no effect on *IL1B* mRNA (Figure 1B). Similar results we obtained from total PBMCs cultured in cell-repellent plates (Figures 1C,D). Total PBMCs cultured in [spn + AAT] showed a lower release of IL-1β in response to LPS without change in *IL1B* mRNA.

### Adherent peripheral blood mononuclear cells cultured in supernatants from heterologous peripheral blood mononuclear cells pre-treated with alpha-1-antitrypsin (Zemaira^®^) release less IL-1β in response to lipopolysaccharide

**FIGURE 2:**
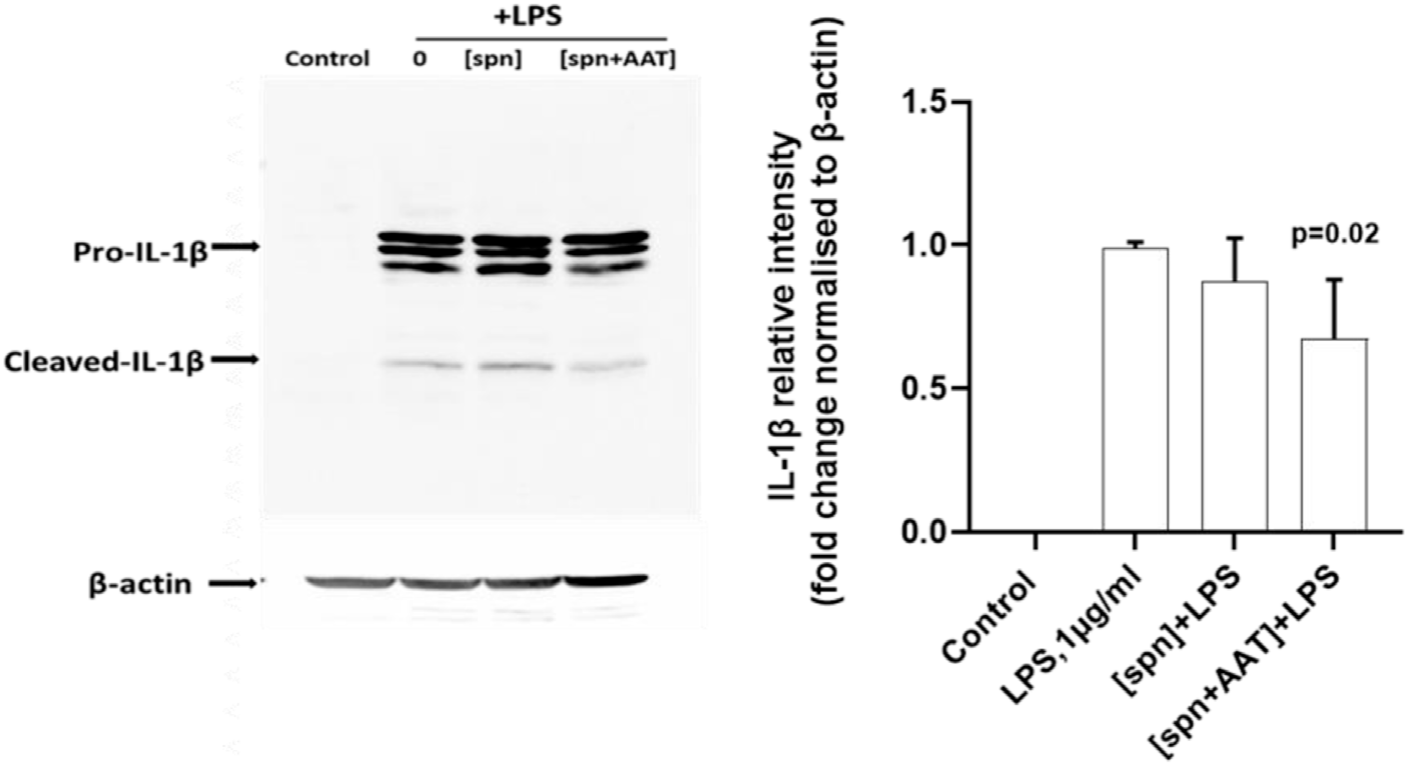
Analysis of intracellular IL-1β in total PBMCs by western blotting shows that supernatants from AAT-pretreated PBMCs [spn + AAT] reduce the accumulation of intracellular pro-IL-1β. Total PBMCs were stimulated with LPS (1 μg/ml) in a regular medium or in supernatants from autologous PBMCs pre-incubated alone [spn] or with 4 mg/ml AAT (Zemaira^®^) [spn + AAT] for 30 min at 37°C, 5% CO_2_, and filtrated through 3 kDa centrifugal filters. Fold changes were calculated for each band using the ratio relative to β-actin (loading control), then normalized by the experimental control (LPS-stimulated cells). A representative blot is shown out of four independent experiments. *p*-value shows a significant difference between LPS and [spn + AAT]+LPS.

We next performed similar experiments using PBMCs from heterologous donors. Here, we measured the LPS-induced IL-1β release and *IL1B* mRNA from adherent PBMCs cultured in the low molecular mass (<3 kDa) fraction of supernatants from heterologous PBMCs prepared with [spn + AAT] or [spn] without pre-treatment with AAT (Zemaira^®^, 4 mg/ml). Similarly like in experiments with autologous PBMCs, [spn + AAT] from heterologous PBMCs significantly lowered LPS-induced IL-1β release but showed no change in *IL1B* expression as compared to PBMCs cultured in [spn] or in a regular medium (Figures 1E,F). In contrast to the *IL1B* mRNA data, [spn + AAT] slightly reduced the accumulation of intracellular pro-IL-1β (Figure 2), pointing to a change in the half-life of intracellular pro-IL-1β or to a change in post-translational control of IL-1β release.

### Conditioned media from autologous peripheral blood mononuclear cells pre-treated with alpha-1-antitrypsin (Zemaira^®^) does not affect the lipopolysaccharide-induced release of TNF-α, IL-6, and IL-8 in total peripheral blood mononuclear cells

**FIGURE 3:**
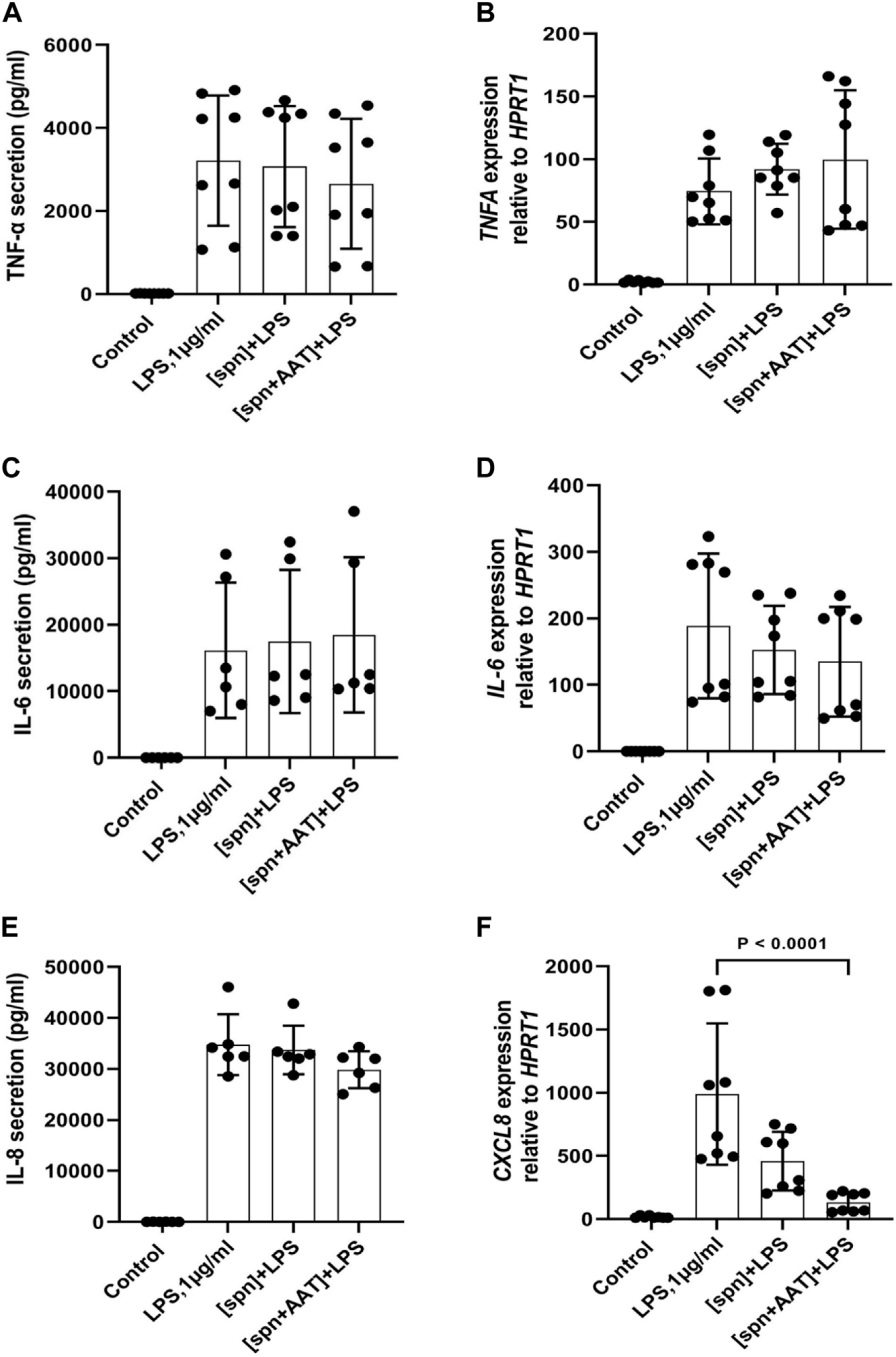
Effects of conditioned media from autologous PBMCs pre-treated with AAT (Zemaira^®^) on LPS-induced release and expression of TNF-α, IL-6, and IL-8 in total PBMCs. LPS-induced TNF-α, IL-6 and IL-8 secretion [**(A,C,E)** respectively] and expression [**(B,D,F)** respectively] within 6 h in total PBMCs cultured in supernatants from autologous PBMCs pre-treated for 30 min with PBS [spn] or with AAT (Zemaira^®^, 4 mg/ml), [spn + AAT]. Each bar represents the mean (SD) from four or three independent donor experiments, with two repeats for each. *p*-value indicates significance between LPS and [spn + AAT]+LPS.

In the same experimental setups as those used for IL-1β release and expression assays, we wanted to investigate if supernatants from PBMCs pre-treated with AAT (Zemaira^®^) also affect the release and/or expression of other cytokines. As presented in Figure 3, total PBMCs in response to LPS significantly increased the release and expression of TNFα, IL-8, and IL-6. However, the low molecular mass fraction of supernatants from PBMCs pre-treated with AAT (Zemaira^®^), [spn + AAT], showed no significant effect on LPS-induced cytokine release. Surprisingly, when compared to cells treated with LPS alone, the same [spn + AAT] significantly lowered *CXCL8/IL8* mRNA (Figure 3). Unfortunately, we were not able to confirm this finding in experiments using supernatants from heterologous donors [IL-8 expression in total PBMCs relative to housekeeping gene *HPRT1*, median (IQR, 25th–75th percentile)]: {[(spn + LPS) 2597 (2597−2449) vs. (spn + AAT) +LPS] 3025 (2770−3805), not significant, *n* = 4 independent donors, with two repeats each]}. The explanation of these discrepancies was out of the scope of this study.

### Conditioned media from peripheral blood mononuclear cells does not contain analyzed pro-resolving inflammatory mediators

We hypothesized that the low melecular mass fraction (<3 kDa) supernatants from PBMCs pre-treated with AAT (Zemaira^®^) may contain small size pro-resolving lipid mediators (SPM), which specifically affect LPS-induced IL-1β release. Therefore, supernatants were analyzed for free oxylipins by means of reversed-phase liquid chromatography coupled with tandem mass spectrometry, and representative and multi-hydroxylated oxylipins were quantified. However, among the plausible candidates investigated, no measurable concentrations of oxylipins (<LLOQ, 0.01–0.1 pmol/L, Supplementary Table S1) were identified. Neither in the filtered supernatants nor in the basal medium, without filtration, we were able to detect analysed oxylipins (hydroxy-PUFA, multiple hydroxy-PUFA among them so called SPM, epoxy-PUFA).

### Total peripheral blood mononuclear cells in response to lipopolysaccharide did not change the release or expression of IL-1β when cultured in filtered supernatants from peripheral blood mononuclear cells pre-treated with alpha-1-antitrypsin preparations other than alpha-1-antitrypsin (Zemaira^®^) or with oxidised (ox) alpha-1-antitrypsin (Zemaira^®^)

**FIGURE 4:**
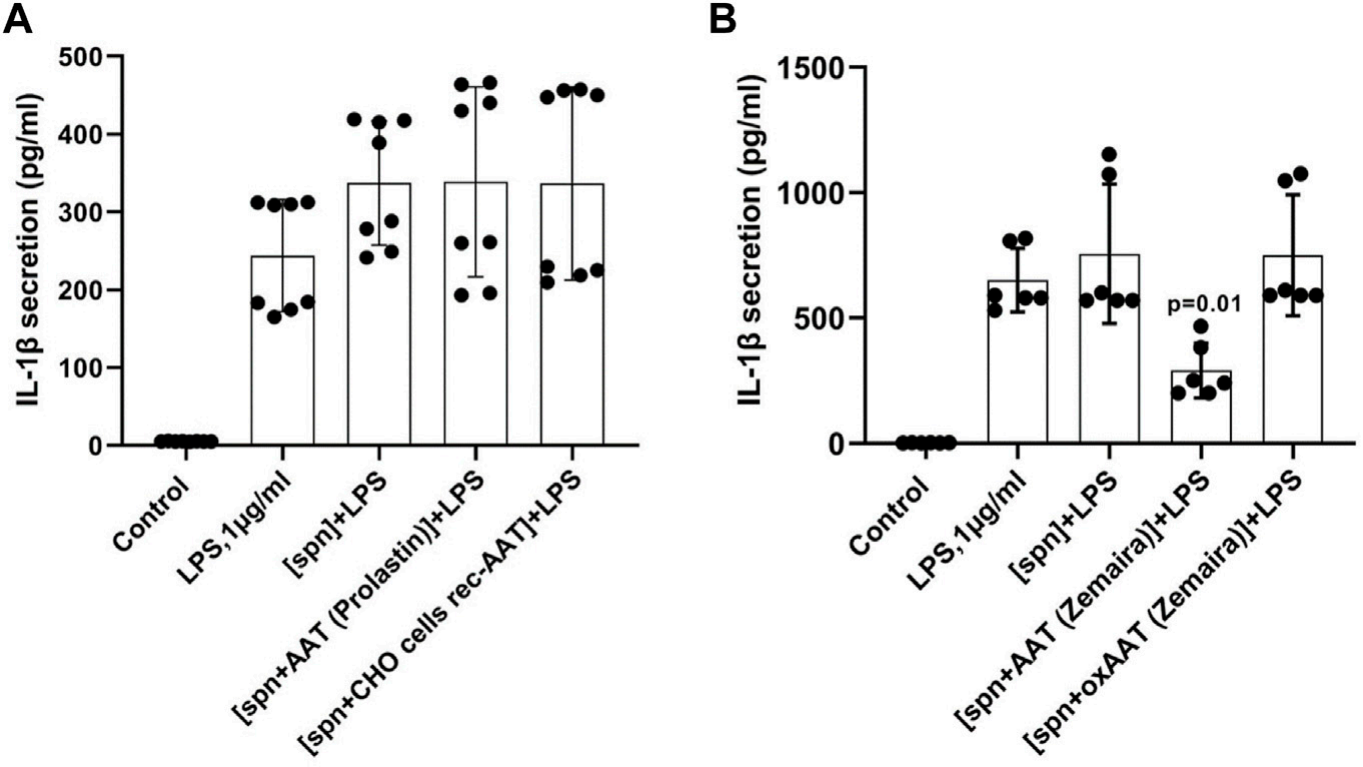
PBMCs in response to LPS did not change the release or expression of IL-1β when cultured in filtered supernatants from PBMCs pre-treated with AAT preparations other than AAT (Zemaira^®^) **(A)** or oxidized AAT (Zemaira^®^) [oxAAT (Zemaira^®^)] **(B)**. **(A)** LPS-induced IL-1β secretion in total human PBMCs cultured for 6 h in supernatants from autologous PBMCs pre-treated for 30 min with PBS [spn] or with AAT (Prolastin^®^) or recombinant human AAT (CHO-recAAT, 4 mg/ml), [spn + CHO cells rec-AAT]. Each bar represents the mean (SD) from four independent donor experiments, with two repeats for each. **(B)** LPS-induced IL-1β secretion in total human PBMCs cultured for 6 h in supernatants from autologous PBMCs pre-treated for 30 min with PBS [spn] or with AAT (Zemaira^®^) or oxidized [ox AAT (Zemaira^®^]. Each bar represents the mean (SD) from three independent donor experiments, with two repeats for each. *p*-value indicates significance between LPS and [spn + AAT (Zemaira^®^)+LPS].

All experiments described above with adherent or total PBMCs were based on the commercial preparation of human plasma AAT (Zemaira^®^). Therefore, we wanted to compare the effects of AAT (Zemaira^®^) with other AAT preparations, namely another commercial preparation of AAT (Prolastin^®^) and recombinant human AAT (CHO-recAAT) produced in CHO cells. For these experiments were empoyed total PBMCs because they contain all IL-1β-producing immune cells, like T cells, B cells, and NK cells, monocytes, and dendritic cells. Unexpectedly, LPS-stimulated total PBMCs, when cultured in supernatants prepared from PBMCs pre-treated with other than AAT (Zemaira^®^) preparations, showed no difference in IL-1β release relative to cells cultured in a regular medium or in a control supernatant (Figure 4A). Similarly, affinity-purified plasma AAT from healthy donors had no effect on the LPS-stimulated IL-1β release (two independent experiments, data not shown). Additional experiments using the same settings revealed that the supernatants of PBMCs pre-treated with oxidized (ox)AAT (Zemaira^®^) also have no effect on the LPS-stimulated IL-1β release (Figure 4B).

### Supernatants from peripheral blood mononuclear cells pre-treated with various alpha-1-antitrypsins as well as alpha-1-antitrypsin proteins per se differ in their total thiol levels

**FIGURE 5:**
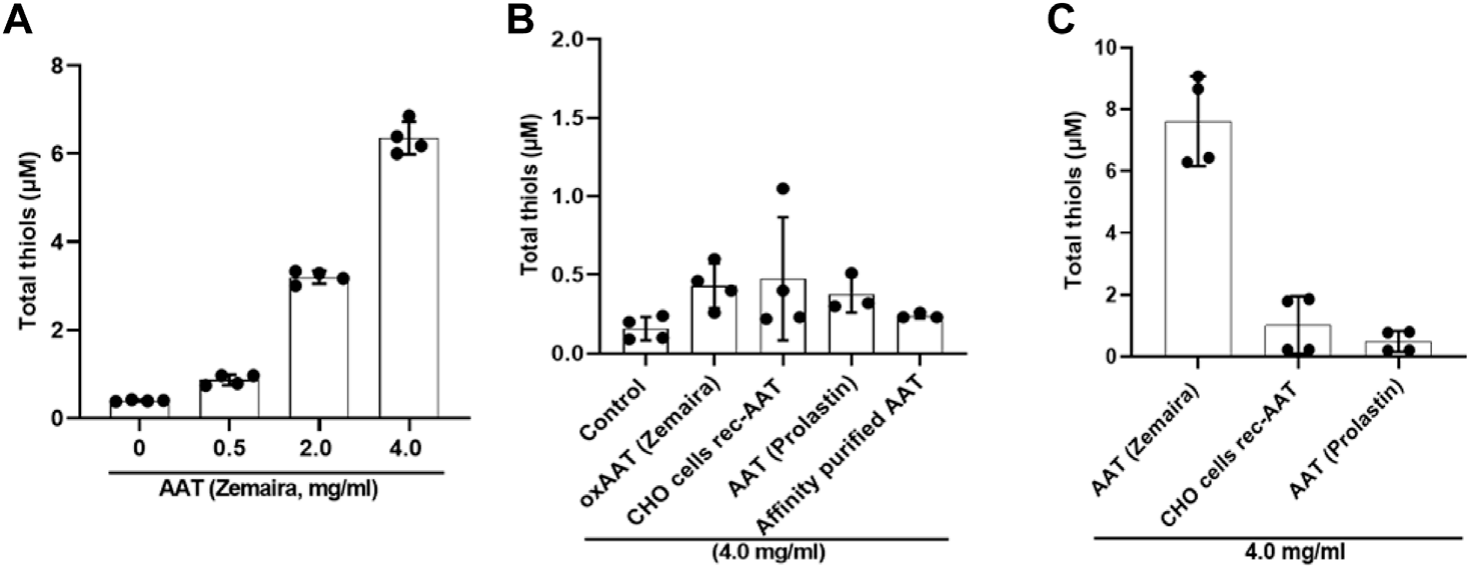
Supernatants from PBMCs pre-treated with various AAT preparations differ in their total thiol levels. Total thiol assay was performed using filtered supernatants (through 3 kDa centrifugal filters) from total PBMCs pre-incubated for 30 min with various concentrations up to 4 mg/ml of AAT **(A)** (Zemaira^®^) or **(B)** with a constant concentration of 4 mg/ml of various AAT preparations, including, oxAAT (Zemaira^®^), and **(C)** with a constant concentration of 4 mg/ml AAT proteins alone prepared in PBS. Each bar represents mean (SD) from two independent experiments with two repeats each. Native AAT (Zemaira^®^) showed concentration-dependent free thiols. Other AAT preparations as well as oxAAT (Zemaira^®^) had only minor levels of free thiols.

An unforeseen finding that PBMCs cultured in filtered supernatants from PBMCs pre-treated with AAT preparations other than AAT (Zemaira^®^) or oxidized AAT (Zemaira^®^) did not lower LPS-induced release of IL-1β raised a question what are the differences between AAT preparations and/or their effects on PBMCs secretome. Among other factors, low-molecular-weight thiols and the redox state of the inflammatory microenvironment are known to affect the expression of inflammatory cytokines (Ghezzi, 2021), inlcuding IL-1β secretion (Tassi et al., 2009). As mentioned earlier, AAT protein has a free thiol (–SH) group on cysteine (Cys232), which can undergo S-nitrosylation and proceed to S-glutathionylation (Xiong et al., 2011; Grek et al., 2013). Some data suggest that the redox state of proteins in the extracellular milieu and on the cell surface, can control the level and bioactivity of IL-1β (Iyer et al., 2009). Hence, in the following experiments, we pre-incubated total PBMCs with various concentrations of AAT (Zemaira^®^) for 30 min, filtered supernatants *via* centrifugal filters (cutoff 3 kDa), and directly used them to determine total thiols. As shown in Figure 5A, total thiol levels in supernatants paralleled with increasing concentrations of AAT (Zemaira^®^). We repeated the total thiol assay with other AAT preparations as well as with artificially oxidized AAT [oxAAT (Zemaira^®^)]. As illustrated in Figure 5B, supernatants from PBMCs treated with oxAAT (Zemaira^®^), AAT (Prolastin^®^), CHO-recAAT or our purified AAT from donor plasma contained very low levels of total thiols. Finally, we measured total thiols in all AAT proteins after buffer exchange to PBS. Indeed, only AAT (Zemaira^®^), but not other preparations of AAT, was positive for free thiols (Figure 5C).

### The cell-free medium containing alpha-1-antitrypsin (Zemaira®) lowers lipopolysaccharide-induced IL-1β release and expression

**FIGURE 6:**
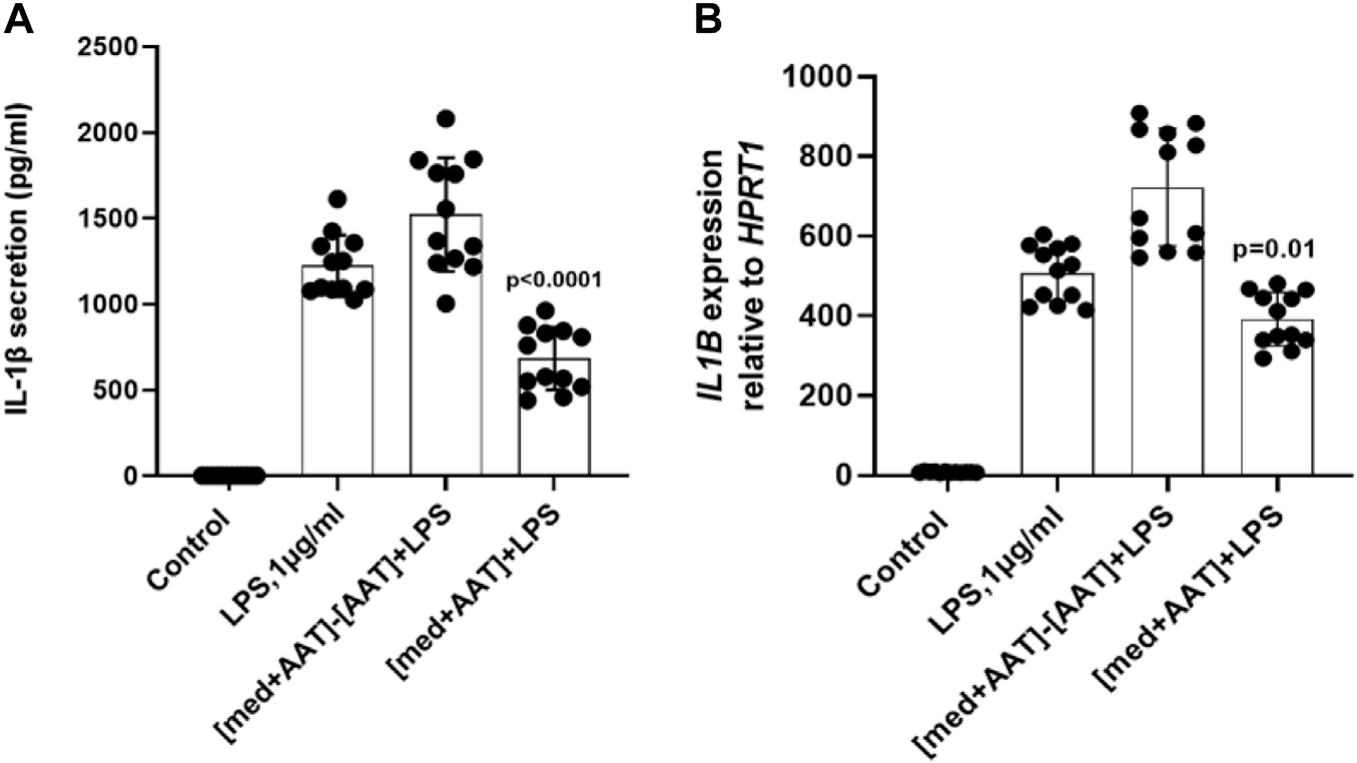
Total PBMCs when cultured in medium containing AAT (Zemaira^®^) show lower IL-1β release and expression in response to LPS. LPS-induced IL-1β secretion **(A)** and *IL1B* mRNA expression **(B)** in total human PBMCs cultured for 6 h in basal medium pre-incubated for 30 min at 37°C, 5% CO_2_, alone or with 4 mg/ml AAT (Zemaira^®^) and added to the cells without [med + AAT] or with filtration through 3 kDa centrifugal filters [med + AAT]−[AAT]. Each bar represents the mean (SD) from three to five experiments with independent donors, two repeats for each.

We next pre-incubated AAT (Zemaira^®^, 4 mg/ml) for 30 min in a cell-free culture medium (without PBMCs) and the medium was when filtrated or not *via* centrifugal filters (cutoff 3 kDa). Both medium preparations- one filtrated to remove AAT and the other containing AAT protein- were used to culture total PBMCs in the presence of LPS for 6 h. As shown in Figures 6A,B, the medium containing AAT significantly lowered LPS-induced IL-1β release and expression, whereas the cell-free medium after removing AAT had no effect on IL-1β release or *IL1B* mRNA expression as compared to LPS-stimulated cells in a regular medium. These results allowed excluding the possibility that the AAT (Zemaira^®^) *per se* contains small molecules, which are lowering the LPS-induced IL-1β release.

### The alpha-1-antitrypsin (Zemaira®) contains more reduced Cys232 than alpha-1-antitrypsin (Prolastin®)

Due to incomplete tryptic digestion, four different peptides (LGMFNIQHCK, LGMFNIQHCKK, RLGMFNIQHCK, RLGMFNIQHCKK) containing Cys232 were identified by the MaxQuant analysis and these cleavage-variants were detected as four different modification-variants, each: 1) without modification (w/o), 2) with oxidation of methionine at position 226 (Ox-Met226), 3) with carbamidomethylation of cysteine at position 232 (CAM-Cys232), and 4) with both, Ox-Met226 and CAM-Cys232. The automated MaxQuant analysis provided an overall calculated value for carbamidomethylation of Cys232 as “ratio mod/base” for AAT (Zemaira^®^) of 5.53 and for AAT (Prolastin^®^) of 0.28 indicating ∼20-fold more carbamidomethylated Cys232 in AAT Zemaira^®^ compared to Prolastin^®^. This finding was supported by our “manual” XIC-based evaluation. For each of the four cleavage-variants of the Cys232-containing peptide of AAT (Zemaira^®^ and Prolastin^®^), we determined the peak area for the four different modification-variants and normalized these values to the highest value that was set to one. Then we determined the mean of each modification variant based on the normalized peak areas from the four values for each cleavage-variant. This analysis revealed that unmodified (w/o) and Met226-oxidized (Ox-Met) peptides were detected at low levels irrespectively of the AAT-source whereas carbamidomethylated peptides (CAM-Cys232 and CAM-Cys232/Ox226-Met) were found at much higher levels in AAT (Zemaira^®^) (Supplementary Figure S1). These findings reveal that carbamidomethylation at Cys232 was considerably more efficient for AAT (Zemaira^®^). As we did not reduce the AAT before carbamidomethylation, our observation suggests an increased degree of reduced Cys232 accessible for carbamidomethylation in AAT (Zemaira^®^) than in AAT (Prolastin^®^).

## Discussion

IL-1β has diverse functionality and significance in health and a broad spectrum of diseases (Kaneko et al., 2019). Under normal conditions, in the absence of cell-activating signals most of the newly synthesized IL-1β is degraded and typically undetectable. The transcription of pro-IL-1β and the release of mature, active IL-1β depend on the cell type and the potency of the cell activating stimuli (Yang et al., 2022). Although IL-1β is an essential coordinator of immune responses and resistance to infections, when released in high levels it exacerbates tissue injuries. Therefore, the mechanisms and agents controlling IL-1β expression and secretion represent a great interest in clinical and experimental research.

Due to the diversity of stimuli, culture conditions, and the cell types secreting IL-1β, the elucidation of the precise mechanisms of IL-1β secretion remains a considerable challenge (Lopez-Castejon and Brough, 2011; Sitia and Rubartelli, 2020). Traditionally, most of the studies focused on the pattern recognition receptor-induced gene expression and inflammasome-mediated cleavage of pro-IL-1β. However, basal redox state of different myeloid cells also contributes to IL-1β production (Carta et al., 2011). The glutathionylation of cysteine in IL-1β by reactive oxygen species and its modulation by the glutaredoxin 1 is another mechanism proposed to control IL-1β levels (Zhang et al., 2017).

Among reported inhibitors of IL-1β release are phospholipase inhibitors (Franchi et al., 2009), anion transport inhibitors (Perregaux et al., 1996), alkylating agents (Laliberte et al., 1999), redox-active drugs (Tassi et al., 2010), and sphingomyelinase inhibitors (Bianco et al., 2009). Numerous studies in animal models *in vivo* and in cell experiments *in vitro* found that human AAT regulates of IL-1β levels (Janciauskiene et al., 2004; Aggarwal et al., 2016; Elshikha et al., 2016; Jeong et al., 2019; Agné et al., 2021). So far, extracellular ATP acting *via* the P2X7 receptor is among the best-known stimuli to induce the inflammasome-dependent release of active IL-1β (Le Feuvre et al., 2002). We found that AAT inhibits ATP signaling and thereby, maturation and release of IL-1β by human leukocytes (Siebers et al., 2018). Moreover, we proposed a signaling pathway triggered by AAT, which involves scavenger receptor CD36 and calcium-independent phospholipase A2β-dependent release of low molecular weight activators of nicotinic acetylcholine receptors leading to the inhibition of the P2X7 receptor that is responsible for inflammasome activation and IL-1β release. The nature of low molecular weight factor(s) released from leukocytes after exposure to AAT remained unknown though we speculated that these factors most probably belong to the metabolites of phosphatidylcholines.

To continue the search for small cellular factor(s) triggered by AAT, we plated human PBMCs in a low molecular mass fraction (<3 kDa) supernatants obtained from autologous or heterologous PBMCs, which were pre-treated or not with AAT preparations. The cultures of PBMCs we exposed to LPS for 6 h and assessed for IL-1β release and *IL1B* mRNA expression. As mentioned above, mechanisms regulating IL-1β secretion are dependent on the cell type. Thus, in contrast to murine macrophages and myelomonocytic cell lines, primary human monocytes after triggering toll-like receptors do not require a second signal for processing and secretion of IL-1β (Piccini et al., 2008). In accordance, adding LPS to human PBMCs was enough to increase significantly *IL1B* mRNA, pro-IL-1β formation, and IL-1β release. To avoid serum AAT and antioxidant effects, we used serum-free conditions in all experiments.

Our initial data supported the notion that supernatants generated from PBMCs pre-treated with human plasma purified AAT (Zemaira^®^) contain small molecular weight factor(s), which significantly lower LPS-induced IL-1β release from total or adherent PBMCs. Total PBMCs include lymphocytes, monocytes, and dendritic cells whereas adherent PBMCs are mostly composed of monocyte/macrophages and dendritic cells. Therefore, the effect of small molecular weight factor(s) on LPS-induced IL-1β release does not seem to be related to specific cell fraction. Though these low-molecular mass factors released from autologous or heterologous PBMCs lowered LPS-induced IL-1β release, they had no effect on LPS-induced release of other cytokines (TNFα, IL-6, and IL-8). Filtered supernatants from PBMCs-pre-treated with AAT had no effect on LPS-induced *TNFA* and *IL6* mRNA expression but also on *ILB* mRNA. Noticeably, supernatants from autologous, but not from heterologous PBMCs treated with AAT, significantly lowered LPS-induced *IL8* expression. Because IL-1β affects IL-8 expression (Naruke al., 2021), one can only speculate that baseline differences in LPS-induced IL-1β levels between cells cultured in supernatants from heterologous and autologous PBMCs might determine these divergent effects on *IL8* expression. Further research is needed to address this issue.

Earlier studies provided evidence that IL-1β, differently from IL-6 and TNFα, may follow a non-conventional route of secretion (Rubartelli et al., 1990), and that multiple mechanisms may be involved in the release of IL-1β within the same population of cells (Lopez-Castejon and Brough, 2011). For example, the treatment of macrophages with LPS can induce the recruitment of IL-1β to autophagosomes. Therefore, the activation of autophagy can lead to the degradation of sequestered IL-1β, and only a rescued fraction of IL-1β will be released (Harris et al., 2011). On the other hand, it has been described that the specialized pro-resolving lipid mediators (SPM), small molecular size bioactive molecules, like resolvins, maresins, and protectins derived from ω-3 polyunsaturated fatty acids (PUFAs), and lipoxins derived from ω-6 PUFAs, express anti-inflammatory effects, which include the inhibition of cytokine production (Chavez-Castillo et al., 2021; Lee, 2021). SPMs seem to activate autophagy in different cells (Prieto et al., 2015; Recchiuti et al., 2020). Although the formation, signaling, and occurrence of SPMs are a matter of debate (Schebb et al., 2022), we thought that SPMs might be these unknown small molecules released from AAT-pre-treated PBMCs, which inhibit the LPS-induced IL-1β release. Hence, we performed a comprehensive analysis of the supernatants for SPMs using a state-of-the-art LC-MS/MS method (Kutzner et al., 2019). However, none of the analyzed supernatants contained quantifiable levels of SPM, nor other oxylipins. Hence, we concluded that oxylipid levels in filtered supernatants are lower than 10–100 pmol/L (Supplementary Table S1) and not sufficient to elicit an effect on LPS-induced IL-1β release.

Reactive oxygen/nitrogen species can cause oxidative damage to biomolecules, contributing to the LPS-induced inflammation, oxidative stress, and IL-1β release (Dong et al., 2021). Extensive research has shown that different cells, including immune cells, secrete redox proteins, which can detoxicate free radicals, and change cellular responses to inflammatory stimuli (Lorenzen et al., 2021). For instance, natural antioxidant glutathione, a tripeptide with an active thiol group can be secreted by specific transporters, and its levels in cell supernatants or biological fluids can range between 2 and 20 µM (Jones and Go, 2011). The same is true for the cysteine/cystine, which is also found extracellularly albeit in lower levels than glutathione (Jones, 2006). Redox regulation involves the conversion of reactive thiols on specific cysteine residues from reduced to oxidized forms. These thiol modifications include glutathionylation, sulfenic acid formation, nitrosylation, and disulfide bond formation. AAT has a reactive thiol group, which can participate in thiol-disulfide interchange reactions. Indeed, historical studies discovered serum complexes of AAT with Bence Jones proteins of the kappa-type formed through thiol-disulfide interchange (SH-SS) (Laurell and Thulin, 1975). Since free radicals play a critical role in activating inflammasome and IL-1β release (Harijith et al., 2014), the thiol-based antioxidants are considered as protectors against oxidative stress and IL-1β over production, we speculated that reactive free thiol group of AAT protein might participate in thiol-disulfide interchange reactions and thus lower LPS ability to induce IL-1β secretion. In fact, filtered supernatants (cutoff 3 kDa) from AAT (Zemaira^®^)-pretreated PBMCs contained about 7 µM of total thiols, and it is plausible, that these thiols might be responsible for the lowering effect of LPS on IL-1β release. Likewise, a glutathione seems to provide the defense mechanism against oxidative stress during P2X7 receptor activation (Park and Kim, 2020), and therefore the effect of AAT on LPS-mediated P2X7 receptor activation, oxidative stress and IL-1β release might depend on the thiol levels. To our surprise, supernatants from PBMCs pretreated with AAT (Prolastin^®^), recombinant CHO expressed AAT, or plasma AAT purified in our laboratory, showed only minor levels of total thiols relative to those measured in supernatants from PBMCs pre-treated with AAT (Zemaira^®^). Concomitantly, supernatants from PBMCs pre-treated with other AAT preparations showed no effect on LPS-induced IL-1β release.

The unexpected finding that only supernatants with AAT (Zemaira^®^)-pre-treated PBMCs contained thiols and lowered LPS-induced IL-1β secretion led us to examine purified AAT preparations by the thiol assay, which also determines free cysteine residues. In fact, only AAT (Zemaira^®^), but no other tested preparations of AAT, was positive for the thiols. As predicted, oxidized AAT (Zemaira^®^) showed only traces of thiols and supernatants from PBMCs pre-treated with oxidized AAT had no effect on LPS-induced IL-1β release. In contrast to other AAT preparations, the treatment with the reducing agent dithiothreitol is an early step in the purification of human AAT (Zemaira^®^) (Boerema et al., 2017). Dithiothreitol is expected to chemically reduce AAT at Cys232, which would remove a covalently bound cysteine group from Cys232, a modification that suggested to be irrelevant for the anti-protease function of AAT (Griffiths and Cooney, 2002). However, according to our results, this latter modification in AAT protein seems to be of relevance in controlling LPS-induced IL-1β release from PBMCs. It is important to point out that, in contrast, not a native but oxidised AAT (Zemaira^®^) inhibited the ATP-induced P2X7 receptor-mediated inflammasome activation and IL-1β secretion (Agné et al., 2021). These divergent results support a notion that a redox-dependent regulation of IL-1β secretion by AAT might depend on the cell type as well as on the stimuli present in the cell microenvironment (Carta et al., 2011). For example, divergences have been reported between inflammasome assembly and IL-1β secretion in THP-1 cells and primary monocytes. Specifically, exposure to reducing agents and down-modulation of thioredoxin resulted in opposite effects on the IL-1β secretion in human primary monocytes and THP-1 cells (Tassi et al., 2009; Zhou et al., 2010).

Taken together our data provide evidence that certain activities of AAT, such as a regulation of IL-1β release, may depend on the availability of free Cys232 in AAT protein. The cysteine oxidation can cause changes within AAT conformational and functional properties (Janciauskiene, 2020), as it has also been shown for other proteins (Hanschmann et al., 2013; Sies and Jones, 2020). Free-thiol containing proteins, such as AAT, may have a crucial significance in the regulation of cytokine-mediated inflammatory processes. Since AAT is used as an augmentation therapy, it is of importance to investigate thoroughly not only anti-protease but also immunomodulatory activities of AAT, which may depend on the status of the Cys232.

## Data Availability

The original contributions presented in the study are included in the article/Supplementary Materials, further inquiries can be directed to the corresponding author.
