# Supplementary material for: Indirect effect of alpha-1-antitrypsin on endotoxin-induced IL-1β secretion from human PBMCs
Source: Front Pharmacol. 2022 Sep 30;13:995869. doi: 10.3389/fphar.2022.995869 (PMC9564231; doi:10.3389/fphar.2022.995869)
Supplement: Supplementary file 1 [file Presentation1.pdf]

Supplementary Table 1. Analyzed oxylipins with their corresponding LOQ (limit of quantification).

Oxylipin	**LLOQ [nmol/l]**
hydroxy fatty acids	
9-HODE	0.0434
13-HODE	0.0313
5-HETE	0.00438
12-HETE	0.0313
15-HETE	0.0275
5-HEPE	0.00738
12-HEPE	0.0125
15-HEPE	0.0125
4-HDHA	0.0125
7-HDHA	0.0125
14-HDHA	0.0172
17-HDHA	0.106
multi-hydroxylated fatty acids	
5(*S*),15(*S*)-DiHETE	0.0125
8(*S*),15(*S*)-DiHETE	0.158
LxA4	0.0313
6(*S*)-LxA4	0.125
15(*R*)-LXA4	0.0313
LxB4	0.125
LxA5	0.125
RvE1	0.0625
RvE2	0.250
18(*S*)-RvE3	0.625
RvE3	0.625
RvD1	0.0313
17(*R*)-RvD1	0.0313
RvD2	0.250
RvD3	0.0625
17(*R,S*)-RvD4	0.0313
RvD5	0.0313
MaR1	0.0625
7-*epi*-MaR1	0.0625
MaR2	0.0313
PDx	0.0491
epoxy fatty acids	
12(13)-EpOME	0.00463
15(16)-EpODE	0.0231
14(15)-EpETrE	0.0313

17(18)-EpETE	0.0938
19(20)-EpDPE	0.0625
dihydroxy fatty acids	
12,13-DiHOME	0.00363
15,16-DiHODE	0.0567
14,15-DiHETrE	0.00313
17,18-DiHETE	0.0138
19,20-DiHDPE	0.0625

Supplementary Figure 1

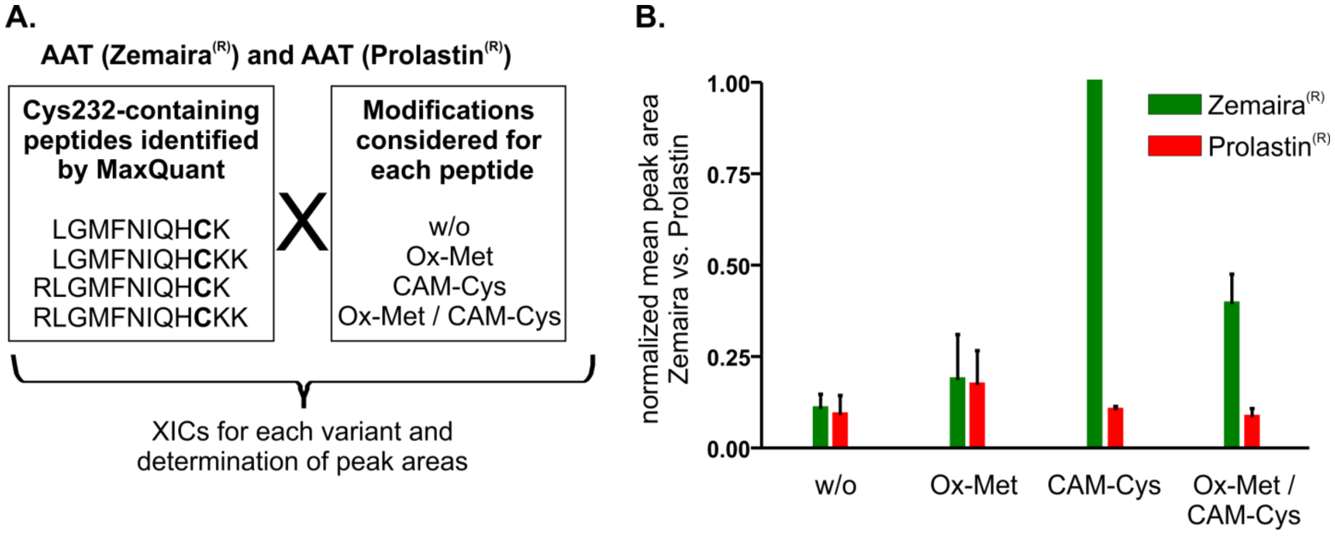

Suppl. Fig 1. Quantification of Cys232 carbamidomethylation in IAA-treated AAT Zemaira® and Prolastin®. (A). Extracted ion chromatograms (XICs) were generated based on monoisotopic m/z values of four Cys232-containing peptides appearing at four different modification states, each (as indicated) and peak areas were determined. (B). Normalized mean peak area depending on the modification state comparing AAT Zemaira® and AAT Prolastin®. For each peptide intensities of differentially modified forms were normalized to the highest value, which was set to 1. Of these normalized intensities for each type of modification a mean value was calculated from the four different peptides.
